# Neuronal pentraxins as biomarkers of synaptic activity: from physiological functions to pathological changes in neurodegeneration

**DOI:** 10.1007/s00702-021-02411-2

**Published:** 2021-08-30

**Authors:** Nerea Gómez de San José, Federico Massa, Steffen Halbgebauer, Patrick Oeckl, Petra Steinacker, Markus Otto

**Affiliations:** 1 Department of Neurology, University of Ulm, Ulm, Germany; 2 Department of Neuroscience, Rehabilitation, Ophthalmology, Genetics, Maternal and Child Health (DINOGMI), University of Genoa, Genoa, Italy; 3 German Center for Neurodegenerative Diseases (DZNE E.V.), Ulm, Germany; 4 Department of Neurology, Martin-Luther-University Halle-Wittenberg, Ernst-Grube-Str. 40, 06120 Halle (Saale), Germany

**Keywords:** Neuronal pentraxin, Biomarker, Synapse, Synaptic function, Neurodegeneration, Cerebrospinal fluid

## Abstract

The diagnosis of neurodegenerative disorders is often challenging due to the lack of diagnostic tools, comorbidities and shared pathological manifestations. Synaptic dysfunction is an early pathological event in many neurodegenerative disorders, but the underpinning mechanisms are still poorly characterised. Reliable quantification of synaptic damage is crucial to understand the pathophysiology of neurodegeneration, to track disease status and to obtain prognostic information. Neuronal pentraxins (NPTXs) are extracellular scaffolding proteins emerging as potential biomarkers of synaptic dysfunction in neurodegeneration. They are a family of proteins involved in homeostatic synaptic plasticity by recruiting post-synaptic receptors into synapses. Recent research investigates the dynamic changes of NPTXs in the cerebrospinal fluid (CSF) as an expression of synaptic damage, possibly related to cognitive impairment. In this review, we summarise the available data on NPTXs structure and expression patterns as well as on their contribution in synaptic function and plasticity and other less well-characterised roles. Moreover, we propose a mechanism for their involvement in synaptic damage and neurodegeneration and assess their potential as CSF biomarkers for neurodegenerative diseases.

## Introduction

Synapses are specialised neuronal structures that transmit nervous impulses between neurons. Synaptic transmission is fundamental to neural functions, including memory formation and learning (Südhof 2018). Synapse dysfunction is emerging as one of the earliest pathological events in many neurodegenerative disorders, and may precede neuronal loss (Gong and Lippa 2010; Janezic et al. 2013; Lleó et al. 2019; Lui et al. 2016; Marttinen et al. 2015; Masliah et al. 2001). Referred to as synaptopathies, these neurodegenerative disorders are characterised by a certain degree of synaptic dysfunction and deregulation of pre- and post-synaptic proteins (Lepeta et al. 2016). The aberrant accumulation of misfolded proteins and other pathophysiological processes common to many neurodegenerative diseases, such as neuroinflammation and excitotoxicity, promote synapses dysfunction and loss. Furthermore, synapses may promote the spread of aberrant proteins via prion-like mechanisms, thus enhancing neurodegeneration (Camporesi et al. 2020; Gong and Lippa 2010; Swanson et al. 2018). In addition, studies in Alzheimer’s disease (AD) found synapse loss as the pathological mechanism that best correlates with cognitive impairment (Terry et al. 1991). Thus, the ability to reliably recognise and follow synaptic damage would provide valuable knowledge on diagnosis, prognosis, staging and therapeutic response in patients. In consequence, an accurate fluid biomarker of synaptic dysfunction would be of great value for the field and may improve the patient’s quality of life by making diagnosis faster and more accurate.

Several studies have provided data on already established [α-synuclein, neurogranin, synaptosomal-associated protein 25 (SNAP-25), synaptotagmin-1 and neuromodulin (GAP-43)] and emerging fluid biomarkers of synaptic damage [β-synuclein, synaptic vesicle glycoprotein 2A (SV2A)] (Camporesi et al. 2020; Halbgebauer et al. 2020; Oeckl et al. 2020). Among the latter, neuronal pentraxins (NPTXs) have gained attention as they participate in synaptic plasticity (O’Brien et al. 2002, 1999; Sia et al. 2007; Xu et al. 2003) and are expressed in brain regions that are primarily affected by AD and other neurodegenerative pathologies (Hawrylycz et al. 2012; Uhlén et al. 2015). NPTXs might be a selective marker for the imbalance of pyramidal neuron-interneuron microcircuits, thought to underlie cognitive impairment in AD (Xiao et al. 2017). Moreover, their structural homology with pentraxins involved in the immune system (Wang et al. 2020) could indicate a role in neuroinflammation and complement-mediated synaptic pruning (Kovács et al. 2020), mechanisms involved in neurodegeneration (Swanson et al. 2018; Tenner et al. 2018). In addition, NPTXs are present in both pre-and post-synaptic compartments providing information about the status of both domains (Xu et al. 2003). Taken together, these features highlight the potential of NPTXs as markers of structural and functional synaptic deficiency in neurodegeneration.

Recent studies in cerebrospinal fluid (CSF) revealed altered NPTXs levels in several neurodegenerative disorders. Even though AD patients have been mostly investigated so far (Begcevic et al. 2018; Brinkmalm et al. 2018; Duits et al. 2018; Galasko et al. 2019; Hendrickson et al. 2015; Lim et al. 2019; Llano et al. 2017; Nilsson et al. 2021; Ringman et al. 2012; Soldan et al. 2019; Spellman et al. 2015; Swanson et al. 2016; Wildsmith et al. 2014; Xiao et al. 2017; Yin et al. 2009), some data are also available for other neurodegenerative disorders such as frontotemporal lobar degeneration (FTLD) and synucleinopathies, as well as for psychiatric disorders (Barschke et al. 2020; Boiten et al. 2020; Charbonnier-Beaupel et al. 2015; Eastwood and Harrison 2010; Moran et al. 2008; Remnestål et al. 2020; van der Ende et al. 2020, 2019). To date, measurements of NPTXs can only be performed in CSF samples, due to the insufficient assay sensitivity for a reliable quantification in blood. Given that lumbar puncture is an invasive procedure, longitudinal studies are missing. Moreover, the lack of consistency between studies may be the result of different inclusion and exclusion criteria and the absence of standardised analytical methods. Thus, prospective studies are required to drive further conclusions about the potential of these proteins as CSF and blood biomarkers of neurodegeneration.

In this review, we summarise the current knowledge about structure and expression pattern of the neuronal pentraxin family and discuss its physiological role in synaptic plasticity and its hypothetical involvement in pathological synaptic degeneration. Finally, we assess their potential as CSF biomarkers and drug targets for different neurodegenerative disorders and give some insights into their speculative role in other diseases of the central nervous system (CNS).

## Neuronal pentraxins: a subfamily of highly conserved pentraxins

**Fig. 1:**
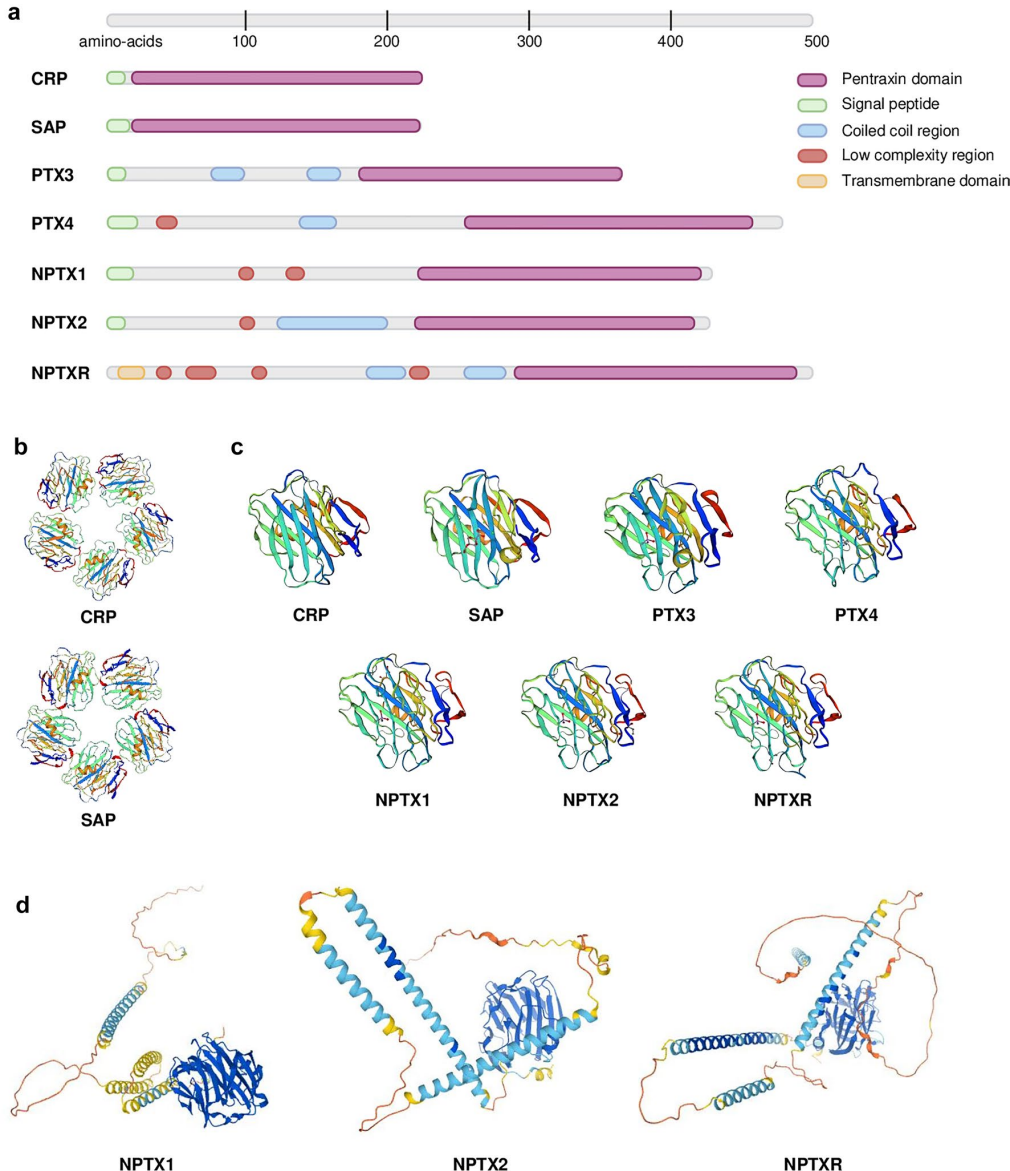
**a** Sequence localisation of the protein domains of pentraxins: pentraxin domain (purple), signal peptide (green), coiled coil region (blue), low complexity region (red) and transmembrane domain (orange). Long pentraxins (PTX3—P26022, PTX4—Q96A99, NPTX1—Q15818, NPTX2—P47972 and NPTXR—O95502) share the C-terminal pentraxin domain with short pentraxins (CRP—P02741 and SAP—P02743), but they contain a long N-terminal domain. Data obtained from Proteomics DB and UniProt. Created with Biorender.com **b** Experimental quaternary structure of the pentameric human CRP and SAP obtained by X-Ray diffraction. Data obtained from the Swiss-Model database (Emsley et al. 1994; Shrive et al. 1996; Waterhouse et al. 2018) **c** Tertiary structure of the pentraxin domain of all members of the pentraxin family. Experimental tertiary structure of the pentraxin domain of CRP, SAP and NPTX1 obtained by X-Ray diffraction, two-layered β sheet with a flattened jellyroll topology (Emsley et al. 1994; Shrive et al. 1996; Suzuki et al. 2020). Predicted structure of the pentraxin domain of PTX3, PTX4 and neuronal pentraxins (NPTX1, NPTX2 and NPTXR) by sequence homology with NPTX1. The pentraxin domain is highly conserved within this family of proteins. Data obtained from the Swiss-Model database. **d** Tertiary structure of the NPTXs obtained with the Protein Structure Database AlphaFold (Senior et al. 2020). The colour code indicates the per-residue confidence score (0–100): dark blue (> 90, very high), light blue (90–70, confident), yellow (70–50, low), orange (< 50, very low)

NPTXs are a family of three proteins (neuronal pentraxin 1 (NPTX1), neuronal pentraxin 2 (NPTX2) and neuronal pentraxin receptor (NPTXR)), part of the pentraxin family. Pentraxins are an evolutionary and highly conserved family of proteins from invertebrates to humans, which highlights their important role in complex organisms. They are humoral pattern recognition receptors that modulate the crossroad between innate and adaptive immune responses (Garlanda et al. 2005). They are defined by a C-terminal pentraxin domain, consisting of roughly 200 amino acids (AA), with a 8 AA structural motif known as the pentraxin sequence (His-X-Cys-X-Ser/Thr-Trp-X-Ser, where X is any AA) (Breviario et al. 1992; Wang et al. 2020) (Fig. 1a). Pentraxins can be classified into two groups: (1) short pentraxins [C-reactive protein (CRP) (Whitehead et al. 1983) together with serum amyloid P component (SAP) (Dowton and McGrew 1990)]; and (2) long pentraxins [pentraxin 3 (PTX3) (Lee et al. 1993), pentraxin 4 (PTX4) (Martinez de la Torre et al. 2010) and NPTXs (Dodds et al. 1997; Hsu and Perin 1995; Omeis et al. 1996)]. Short pentraxins are arranged in pentameric structures with a discoid shape (CRP—PDB ID: 1GNH; SAP—PDB ID: 1SAC) (Emsley et al. 1994; Shrive et al. 1996), however the quaternary structure of long pentraxins is unknown (Fig. 1b,c). They share the C-terminal pentraxin domain with short pentraxins, but they contain a long N-terminal domain unrelated to other proteins (Bottazzi et al. 2010) (Fig. 1a–c). In 2021, the publication of AlphaFold 2 enabled the prediction of the three-dimensional structure of NPTXs, among many other proteins (Jumper et al. 2021; Senior et al. 2020) (Fig. 1d). CRP, SAP and PTX3 participate in innate immune responses against extracellular and intracellular pathogens through activation and regulation of the complement cascade, microorganism opsonisation and agglutination. Moreover, they take part in inflammation and clearance of apoptotic cells (Bottazzi et al. 2010). NPTXs modulate synaptic function and plasticity (Xu et al. 2003), however their potential role in the immune system has been poorly described in scientific literature and is open to debate. A new study published in 2021, confirmed a role of NPTXs in synaptic pruning by activation of the complement cascade (Kovács et al. 2020). Accordingly, NPTXs may perform some of the functions of the classical pentraxins in the brain, with additional roles in synaptic function.

### Expression pattern and structure of neuronal pentraxins

#### Neuronal pentraxin 1

**Fig. 2:**
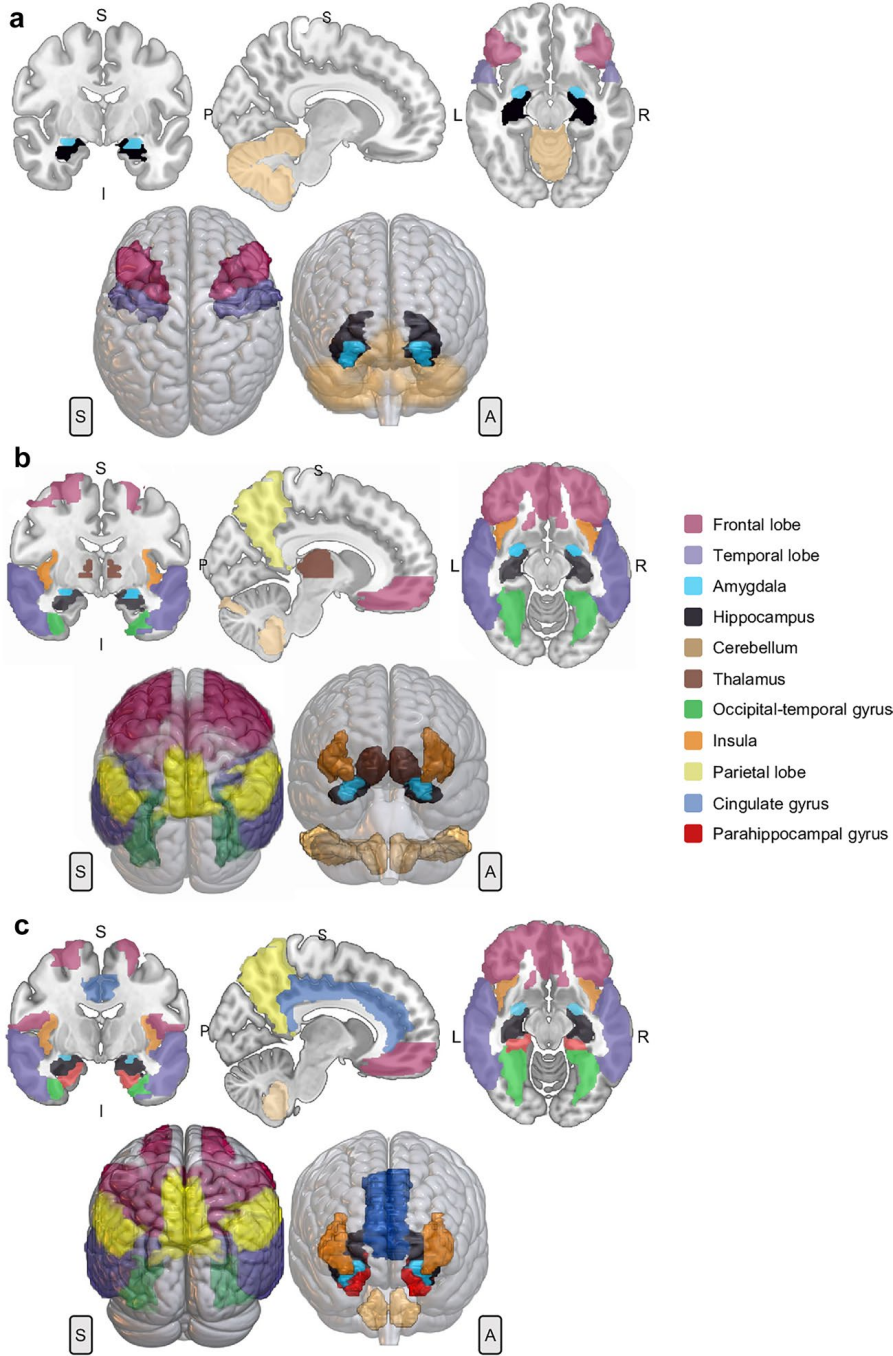
2D representation according to axial, coronal and sagittal axes and 3D rendering of the areas with the highest RNA expression of NPTX1 (**a**), NPTX2 (**b**) and NPTXR (**c**). The gene expression data was obtained from Allen Human Brain Atlas (Hawrylycz et al. 2012). Microarray analysis of 900 anatomically defined sites of 6 patients was conducted with two different probes. The areas with a *z* score > 1 were considered. Regions of interest were obtained by MarSBar toolbox (Brett et al. 2002) to be overlaid to the MNI referential atlas (MRIcroGL software, https://www.nitrc.org/projects/mricrogl) and are represented in different colours, as follows: frontal lobe (wine red), temporal lobe (purple), amygdala (light blue), hippocampus (black), cerebellum (copper), thalamus (brown), occipital-temporal gyrus (green), insula (orange), parietal lobe (yellow), cingulate gyrus (dark blue) and parahippocampal gyrus (red). Anatomical positions: anterior (A), posterior (P), superior (S), inferior (I), left (L) and right (R)

In humans, the *NPTX1* gene is located on chromosome 17q25.3. Its cDNA clone sequence consists of 150 bp 5’UTR, 1.3 kb coding region and 3.6 kb 3′ UTR (Omeis et al. 1996). The coding region is divided in 5 exons and 4 introns (Gene ID: 4884). NPTX1 protein (also known as NP1) (Q15818) is a 47 kDa secreted glycoprotein of 432 AA sequence (Schlimgen et al. 1995). It contains several domains: a peptide signal (1–22 AA), a N-terminal with two low complexity regions (98–108 AA and 129–140 AA) and a C-terminal pentraxin domain (222–428 AA) (Schmidt et al. 2018) (Fig. 1a). NPTX1 has two sites for N-glycosylation (Asn154 and Asn193) and several Cys residues. Three cysteine residues are located in the N-terminal (Cys32, Cys42 and Cys89) and are involved in disulphide bonds mediating the formation of homomeric and heteromeric assembly with NPTX2 (Xu et al. 2003). Cys256 and Cys316 form another disulphide bond within the pentraxin domain (The UniProt Consortium 2021). In 2020, the crystal structure of the pentraxin domain of NPTX1 was obtained by X-Ray Diffraction. The pentraxin domain exhibits the same structure as CRP and SAP, a two-layered β sheet with a flattened jellyroll topology (Suzuki et al. 2020) (Fig. 1c). Since 2021, the full three-dimensional structure can be obtained using the Protein Structure Database AlphaFold (Senior et al. 2020) (Fig. 1d). NPTX1 is mainly expressed in the brain, but also present in the retina and vitreous humour, adrenal gland, rectum, and testis (Schmidt et al. 2018; Uhlén et al. 2015) (NPTX1 data available from v20.1.proteinatlas.org). Within the human brain, it is highly expressed in the cerebral cortex (including hippocampal formation), cerebellum, and amygdala (Fig. 2a). With regards to its cellular localisation in the cerebral cortex, NPTX1 is present in neuronal projections, mainly in the pre-synaptic terminal; in particular, in the mossy fibres projecting from granule cells to the dentate gyrus, in the stratum lucidum of CA3 and stratum radiatum of CA1. Moreover, it has also been detected in neuronal cell bodies, in the neuropil of entorhinal cortex and hippocampus, and in glial cells (Abad et al. 2006; Uhlén et al. 2015) (Table 1).

**Table 1:** Characteristics of the neuronal pentraxins

	NPTX1	NPTX2	NPTXR
Gene			
Genomic localization	17q25.3	7q22.1	22q13.1
Sequence	150 bp 5'UTR, 1.3 kb coding region (5 exons and 4 introns), 3.6 kb 3'UTR	1.3 kb coding region (5 exons and 4 introns), 1.2 kb 3'UTR	3.9 kb 3'UTR, 1.5 kb coding region (5 exons and 4 introns)
Protein			
Name	NPTX1, NP1	NPTX2, NP2, Narp (rat), apexin/p50 (guinea pig)	NPTXR, NPR
Size	47 kDa	47 kDa	53 kDa
Type	Secreted glycoprotein	Secreted glycoprotein	Type-II transmembrane
Sequence	432 AA	431 AA	500 AA
Domains	Peptide signal (1–22 AA), two low complexity regions (98–108 AA, 129–140 AA), pentraxin domain (222–428 AA)	Peptide signal (1–17 AA), coiled coil motif (121–199 AA), low complexity region (96–109 AA), pentraxin domain (219–424 AA)	Intracellular domain (1–7 AA), transmembrane domain (7–29 AA), four low complexity regions (38–52 AA, 58–90 AA, 106–118 AA, 218–228 AA), two coiled coil motifs (188–214 AA, 248–286 AA), pentraxin domain (288–494 AA)
Tissue expression	Brain, retina, vitreous humour, adrenal gland, rectum, testis	Pancreas, vitreous humour, colon, endocrine tissues (pituitary and adrenal glands), brain, testis, prostate gland	Brain, rectum, adrenal glands, helper T-lymphocytes

*NPTX1* neuronal pentraxin 1, *NPTX2* neuronal pentraxin 2, *NPTXR* neuronal pentraxin receptor, *AA* amino acids, *UTR* un-translated region

#### Neuronal pentraxin 2

The human *NPTX2* gene is located on chromosome 7q22.1. Its cDNA clone sequence consists of 1.3 kb coding region and 1.2 kb 3’UTR (Hsu and Perin 1995). The coding region is divided in 5 exons and 4 introns (Gene ID: 4885). NPTX2 protein (also known as NP2, Narp in rat and apexin/p50 in guinea pig) (P47972) is a 47 kDa secreted glycoprotein, 431 AA long (Hsu and Perin 1995; Tsui et al. 1996). It is a calcium-dependent lectin capable of binding its ligands in a calcium-dependent manner (Tsui et al. 1996). It contains similar domains as NPTX1, a peptide signal (1–17 AA), a coiled coil motif (121–199 AA) and a low complexity region (96–109 AA) in the N-terminal and a pentraxin domain (219–424 AA) in the C-terminal (Schmidt et al. 2018) (Fig. 1a). NPTX2 has three sites of N-glycosylation (Asn148, Asn189 and Asn393). All cysteines in the N-terminal are involved in intermolecular disulphide bonds, which are necessary and sufficient to form multimers. Cys29 and Cys41 form the core of NPTX2 hexamer structure, whereas Cys95 intervenes in the interaction between different hexamers. Cys253 and Cys313 form an additional disulphide bond within the pentraxin domain (The UniProt Consortium 2021; Xu et al. 2003). Desheng Xu et al. (2003) proposed a cyclic multimeric structure by homology with short pentraxins (Xu et al. 2003) (Fig. 1b). Until 2021, only the structure of the pentraxin domain could be obtained by sequence homology analysis with NPTX1 (Waterhouse et al. 2018) (Fig. 1c). Nowadays, the Protein Structure Database AlphaFold can predict the full three-dimensional structure of this protein (Senior et al. 2020) (Fig. 1d). NPTX2 is the most broadly expressed of the three NPTXs. It is present in the pancreas, vitreous humour, colon, endocrine tissues (pituitary gland and adrenal gland), brain, testis, and prostate gland. Within the brain, it is mainly expressed in a subset of cells in the anterior pituitary gland and in neurons in the cerebral cortex (Schmidt et al. 2018; Uhlén et al. 2015) (NPTX2 data available from v20.1.proteinatlas.org) (Fig. 2b). Additionally, it was detected in a subpopulation of glial cells, including microglia, in post-mortem tissue of Parkinson’s disease (PD) patients (Moran et al. 2008) (Table 1).

#### Neuronal pentraxin receptor

In humans, NPTXR is located on chromosome 22q13.1. Its cDNA clone sequence consists of 3.9 kb 3′UTR and 1.5 kb coding region (Dodds et al. 1997). The coding region is divided in 5 exons and 4 introns (Gene ID: 23,467). NPTXR (also known as NPR) (O95502) is a 53 kDa type-II transmembrane protein with 500 AA. It is anchored to the plasmatic membrane through the N-terminal (7–29 AA). However, multiple intracellular isoforms are described (Cho et al. 2008). This protein consists of a short intracellular domain (1–7 AA), four low complexity regions in the N-terminal (38–52 AA, 58–90 AA, 106–118 AA, 218–228 AA), two coiled coil motifs (188–214 AA and 248–286 AA) and the pentraxin domain in the C-terminal (288–494 AA) (Schmidt et al. 2018) (Fig. 1a). NPTXR has three sites of N-glycosylation (Asn42, Asn216 and Asn463) and two cysteines, Cys322 and Cys383, which form a disulphide bond within the pentraxin domain (The UniProt Consortium 2021). Until 2021, only the structure of the NPTXR pentraxin domain could be obtained by sequence homology analysis with NPTX1 (Waterhouse et al. 2018) (Fig. 1c). Just like NPTX1 and NPTX2, the full three-dimensional structure of NPTXR can now be obtained using the software AlphaFold (Senior et al. 2020) (Fig. 1d). At present, NPTXR is proposed as the receptor for NPTX1 and NPTX2, due to their binding affinity (Dodds et al. 1997) and NPTXR’s ability to recruit NPTX1 and NPTX2 to the synaptic membrane (Kirkpatrick et al. 2000). NPTXR is mainly expressed in brain in the cytoplasm and synapse of neurons and neuropil, mostly in cerebral cortex and hippocampal formation (Uhlén et al. 2015) (NPTXR data available from v20.1.proteinatlas.org) (Fig. 2c). Outside the CNS, it is localised in rectum, adrenal glands and in helper T-lymphocytes (Schmidt et al. 2018) (Table 1).

### Role of neuronal pentraxins in synaptic function and plasticity

**Fig. 3:**
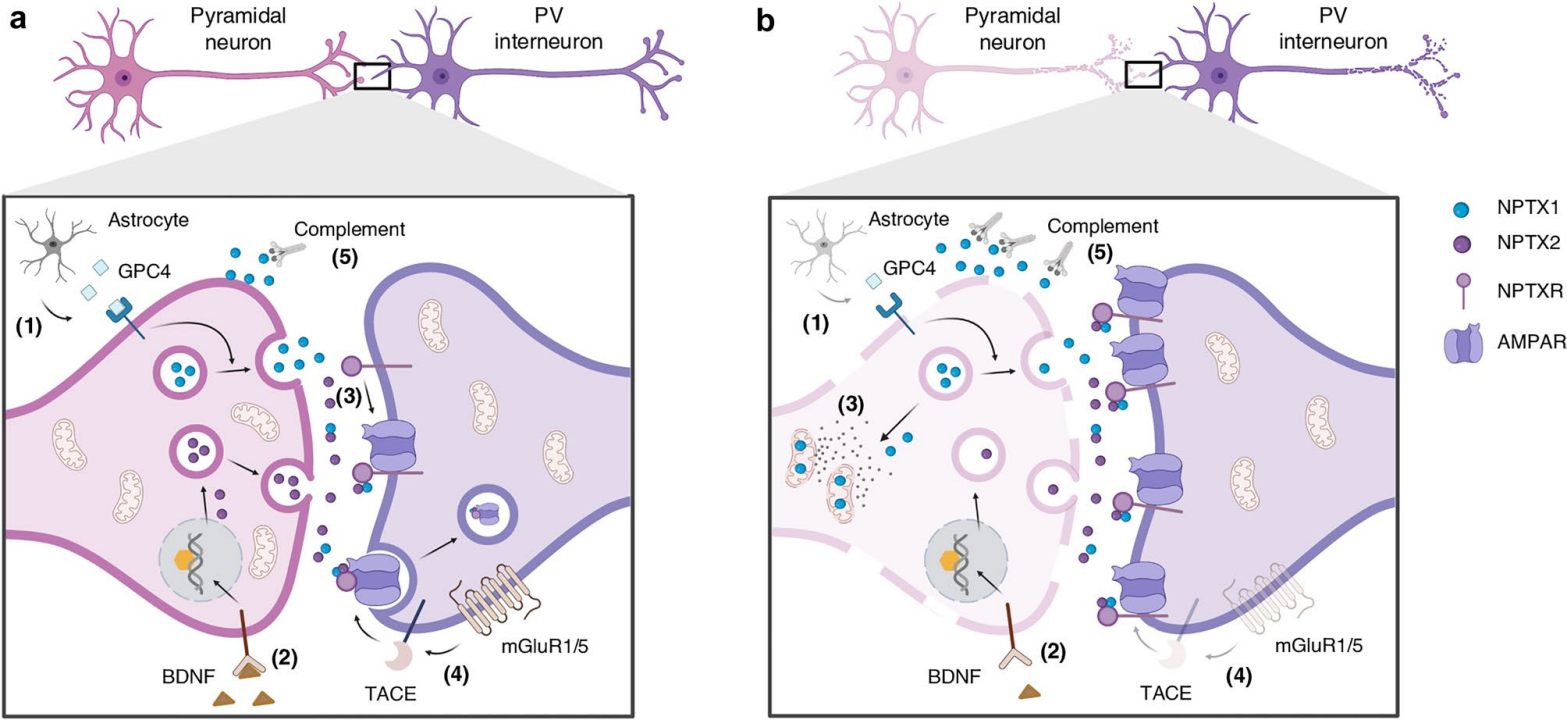
Schematic illustration of the proposed mechanism for the physiological role of neuronal pentraxins (NPTXs) (**a**) and the hypothesised pathological mechanisms in neurodegeneration (**b**). **a** The NPTX1 release from the pre-synaptic terminal into the synaptic cleft may be modulated by the astrocyte-secreted glypican-4 (GPC4) (1), whereas NPTX2 is synthesize and release in response to neuronal activity or BNDF (2). Once in the synaptic cleft, NPTX1 and NPTX2 can form heterocomplexes and bind to NPXR, which is anchored in the post-synaptic membrane. The NPTX complex is then able to cluster and stabilise α-amino-3-hydroxy-5-methyl-4-isoxazolepropionic acid receptor (AMPAR) in the post-synaptic membrane, modulating the excitatory drive onto parvalbumin (PV) interneurons (3). In the presence of excess of glutamate in the synaptic cleft, the mGLUR1/5 receptor is activated and the metalloprotease tumour necrosis factor-alpha converting enzyme (TACE) cleaves the transmembrane domain of NPTXR. Cleaved NPTXR and AMPAR are internalised via endocytosis. This mechanism may protect against excitotoxicity (4). NPTX1 may play a role as activator of the classical complement pathway, leading to microglia-dependent apoptosis (5). **b** Several hypotheses could explain the deregulation of NPTXs and the excess of synaptic loss observed in neurodegeneration. The loss of astrocytic functions and reduced BDNF could lead to a decrease in synthesis and release of NPTX1 and NPTX2, respectively (1, 2). Under low neuronal activity, NPTX1 translocates to the mitochondria, activating BAX-dependent apoptosis and hindering the translocation of mitochondria to the pre-synaptic terminal (3). An alteration of the mechanism to detect excess of glutamate could decrease the endocytosis of NPTX complexes, leading to excitotoxicity (4). The complement-microglia pathway activated by NPTX1 may be intensified upon neurodegenerative processes, favouring the excess synaptic loss observed in these disorders (5). Created with Biorender.com

NPTXs are synaptic organizers located in the synaptic cleft. They are part of the family of extracellular scaffolding proteins, responsible for regulating the assembly of pre- and post-synaptic compartments by recruiting post-synaptic receptors (Yuzaki 2018). NPTXs play a role in synaptogenesis (Farhy-Tselnicker et al. 2017; Figueiro-Silva et al. 2015; Sia et al. 2007), synaptic plasticity (O’Brien et al. 2002, 1999; Sia et al. 2007; Xu et al. 2003), homeostasis (Pribiag and Stellwagen 2014), metabotropic glutamate receptor long-term depression (LTD), synapse elimination (Cho et al. 2008), clearance of synaptic debris and toxins from neurons (Dodds et al. 1997) and neurite outgrowth (Tsui et al. 1996). Moreover, there is evidence that NPTXs are involved in intracellular processes such as mitochondrial dynamics and trafficking, and neuronal apoptosis (Clayton et al. 2012; Rahim et al. 2013; Tseng and Bixby 2011). In addition, recent studies concluded a role of NPTXs in synaptic pruning by activation of the classical complement cascade (Kovács et al. 2020) (Fig. 3a). In humans, they participate in nervous system development (Bjartmar et al. 2006; Boles et al. 2014).

NPTX1 and NPTX2 are the two soluble proteins from the neuronal pentraxin family which colocalise at synapses in the forebrain, including the neocortex (study conducted in rat) (Xu et al. 2003). NPTX1 is constitutively released from excitatory synapses and was initially described for its neuronal activity-independent synaptogenic roles (Kirkpatrick et al. 2000; Xu et al. 2003). Conversely, another study concluded an upregulation of NPTX1 expression upon reduction in neuronal activity, contributing to neuronal cell death (DeGregorio-Rocasolano et al. 2001) and mitochondrial accumulation of the apoptosis regulator BAX (Clayton et al. 2012). Moreover, NPTX1 negatively regulates excitatory synapse density through interaction with a subunit of slow-rectifying-gated Kv7 potassium channel (Figueiro-Silva et al. 2015). NPTX1 is enriched in the synaptic plasma membrane and also detected in mitochondria (in accordance with its role in mitochondrial apoptosis) (Kovács et al. 2020). New data suggested astrocyte-secreted glypican-4 (GPC4) to induce NPTX1 release from pre-synaptic terminals through type 2a receptor protein tyrosine phosphatase (RPTPδ) (Farhy-Tselnicker et al. 2017) (Fig. 3a-1). NPTX2 is an immediate early gene (IEG) and acts in a neuronal activity-dependent manner (Tsui et al. 1996; Xu et al. 2003). As an IEG, it is rapidly and transiently expressed, and belongs to a family of genes that participate in learning, memory, and long-term plasticity (Gallo et al. 2018). It is released from the pre-synaptic terminal in response to neuronal activity, seizure (O’Brien et al. 1999), long-term potentiation (LTP) (Tsui et al. 1996) or brain-derived neurotrophic factor (BDNF) (Mariga et al. 2015). BDNF bidirectionally regulates NPTX2 expression at the transcriptional level, even in the absence of neuronal activity (Fig. 3a-2). BNDF-induced NPTX2 is required for glutamatergic synaptic transmission and LTP in mossy fibres in the dendate gyrus (Mariga et al. 2015; Wibrand et al. 2006). NPTX2 is located in the synaptic cytoplasm and plasma membrane (Kovács et al. 2020).

NPTX1 and NPTX2 are present in both pre- and post-synaptic compartments (Xu et al. 2003). Under physiological conditions, NPTX2 is mainly secreted from the pre-synaptic terminal into the synapses (Chang et al. 2010; Reti et al. 2002). After their release into the synaptic cleft, both soluble pentraxins (NPTX1 and NPTX2) become rapidly associated into heteromultimers. Those heteromultimers require N-terminal protein–protein interactions and specific disulphide bonds, involving Cys29 and Cys95 of NPTX2. The relative ratio of the two proteins in the complex is time- and activity-dependent (Xu et al. 2003). The complex can also interact with NPTXR, which is anchored in the post-synaptic membrane through its N-terminal domain (Dodds et al. 1997) (Fig. 3a-3). NPTXR knock-out (KO) mice presented 50% reduced levels of NPTX1 and NPTX2 in the brain, indicating a possible role of NPTXR as stabilizer of NPTX1 and NPTX2 at synapses (Bjartmar et al. 2006).

The NPTX complex acts as trans-synaptic signalling molecule, regulating the activity of neurotransmitter receptors at excitatory synapses. This is supported by its colocalization with the post-synaptic density protein 95 (PSD-95), a excitatory synaptic scaffolding protein; but not with glutamic acid decarboxylase, a marker of inhibitory axons (Chang et al. 2010; Cho et al. 2008; Xu et al. 2003). In particular, the complex promotes α-amino-3-hydroxy-5-methyl-4-isoxazolepropionic acid receptor (AMPAR) aggregation and stabilisation (O’Brien et al. 1999) (Fig. 3a-3). In vitro studies reported that NPTX2 has a greater effectiveness in clustering AMPAR than NPTX1, due to its N-terminal distinctive properties. In the heteromultimer, NPTX2 enhances NPTX1’s clustering capability, increasing the complex’s ability to aggregate AMPAR and promote synaptogenesis (Xu et al. 2003). The three NPTXs interact, directly or indirectly, with the N-terminal extracellular domain of AMPAR subunits (GluA1, GluA2, GluA3 and GluA4), via their conserved pentraxin domain (Chang et al. 2010; Cho et al. 2008; Lee et al. 2017; Sia et al. 2007; Suzuki et al. 2020). Accordingly, NPTXs act as bimodular proteins. The pentraxin C-terminal domain interacts and promotes AMPAR clustering and stabilisation, whereas the N-terminal coiled coil domain mediates self-association.

NPTXs are enriched at excitatory glutamatergic synapses of pyramidal neurons onto parvalbumin (PV) interneurons in the mice hippocampus (Chang et al. 2010) (Fig. 3a). PV interneurons are the most common interneuron subtype in the hippocampus (Pelkey et al. 2017) and are critical regulators of prefrontal cortex-dependent behavioural responses (Ferguson and Gao 2018). They are GABAergic, fast-spiking interneurons, crucial for network synchronisation of pyramidal neurons (Hu et al. 2014) and the optimal balance of excitation/inhibition (E/I) in cortical circuits. They confer a higher level of computational complexity by modulating pyramidal neuronal gain and preventing excessive excitatory signalling (Ferguson and Gao 2018). Additionally, PV interneurons participate in the generation and maintenance of gamma oscillations, required for working memory (Cardin et al. 2009; Howard et al. 2003). The presence of high-conductance, rapidly gating glutamatergic receptors is needed for neural oscillations. NPTXs regulate circuit rhythmogenesis and integration by controlling expression and synaptic localization of AMPAR and coordination of excitatory synapses maturation onto PV interneurons (O’Brien et al. 1999; Pelkey et al. 2015). NPTX2 KO mice had an overall reduction of pyramidal neuron excitatory drive onto PV interneurons (Gu et al. 2013). In addition, the double KO (*Nptx2*^−/−^, *Nptxr*^−/−^) presented disrupted gamma oscillations and reduction of feedforward inhibition as a result of the reduction of one of the subunits of the AMPAR (Pelkey et al. 2015). The dense perineuronal net (proteoglycan network structure on the cell surface), characteristic of PV interneurons (Carceller et al. 2020; Härtig et al. 1999), is required to stabilise NPTX2 in the synaptic cleft at these synapses (Chang et al. 2010).

NPTXs contribute to various forms of synaptic plasticity. They are not exclusively present in PV interneuron synapses in the hippocampus, as they also regulate early synaptic refinements in retinal ganglion cell projections to dorsal lateral geniculate nucleus (Bjartmar et al. 2006). Moreover, they modulate LTD in synapses formed in hippocampus (Schaffer collateral—CA1 synapses), and cerebellum (Purkinje cells) (Cho et al. 2008). In vitro studies revealed that, in response to the activation of group 1 metabotropic receptor (mGluR1/5), the metalloprotease tumour necrosis factor-alpha converting enzyme (TACE)—implicated in shedding of proteins (Black 2002)—cleaves NPTXR, releasing it from its N-terminal transmembrane domain. Cleaved NPTXR is enabled to form membrane clusters with NPTX2 and is rapidly internalised and colocalizes with AMPAR in post-synaptic endosomes at synapses in the brain (Fig. 3a-4). This mechanism is necessary for LTD in hippocampus and cerebellum (Cho et al. 2008). A potential role of NPTXR as universal organizer of both excitatory and inhibitory synapses was observed in in vitro studies (Lee et al. 2017). However, the role of NPTXs in inhibitory synapses is less explored.

In summary, NPTXs are a family of proteins involved in homeostatic synaptic function and plasticity by recruiting post-synaptic receptors into synapses. Recent findings suggested an opposite regulation of the two soluble NPTXs, NPTX1 and NPTX2, by neuronal activity. NPTX1 is evoked during low neuronal activity and negatively regulates synaptic plasticity at excitatory synapses (DeGregorio-Rocasolano et al. 2001; Figueiro-Silva et al. 2015). Moreover, it participates in synaptic pruning by activation of the classical complement cascade (Kovács et al. 2020) (Fig. 3a-5) and may act as a sensor of harmful stimuli, targeting affected neurons to apoptotic cell death (Clayton et al. 2012; Rahim et al. 2013). In contrast, NPTX2 is activated in response to high neuronal activity and promotes synaptic plasticity at excitatory synapses (Tsui et al. 1996). This opposite role could act as a feedback mechanism to ensure homeostatic synaptic plasticity and precisely regulate the excitatory drive onto PV interneurons. In addition, NPTX heterocomplex play a dual role in synapse formation and depression. During synapse formation, NPTXs may capture and cluster AMPAR in the post-synaptic terminal; whereas during synapse depression, the complex is released from the membrane and captures AMPAR in post-synaptic endosomes. Early in development and in emerging synapses, NPTXs participate in synapse formation by clustering of AMPAR; while in mature synapses or chronic stimulation, they regulate LTD (Cho et al. 2008; O’Brien et al. 1999). This bidirectional mechanism is crucial to provide long-term plasticity to the involved networks.

## Neuronal pentraxins in synaptic dysfunction and neurodegeneration: a proposed mechanism

Synaptic dysfunction and loss might be one of the earliest pathological mechanisms in the course of neurodegeneration (Gong and Lippa 2010; Janezic et al. 2013; Lui et al. 2016; Marttinen et al. 2015; Masliah et al. 2001; Selkoe 2002). The disruption of the protein network at the post-synaptic terminal is crucial for homeostatic synaptic plasticity (Gong and Lippa 2010). In particular, the deregulation of AMPA and/or NMDA receptors in inhibitory interneurons and the dysregulation of E/I balance could be responsible for the cognitive and behavioural abnormalities characteristic of a range of neurodegenerative and psychiatric disorders (Belforte et al. 2010; Bozzi et al. 2018; Caputi et al. 2012; Foss-Feig et al. 2017; Fuchs et al. 2007; Korotkova et al. 2010; Lewis et al. 2005; Maheshwari et al. 2013; Nelson and Valakh 2015; Palop and Mucke 2016; Xu et al. 2020). Moreover, complement-mediated synaptic pruning is essential for the normal function of the CNS, and it is hypothesised that alterations in this system contribute to excess synaptic loss and cognitive impairment in neurodegenerative disorders (Tenner et al. 2018). NPTXs could be involved in some of these pathogenic processes, given their role in AMPAR recruitment, modulation of excitatory synaptic drive and synaptic pruning. Consequently, targeting these proteins may be an effective strategy for the treatment of cognitive impairments.

Changes in the synthesis and/or secretion into the synaptic cleft of NPTX1 and NPTX2 could result in the impairment of their mode of action. If the role of astrocytes in NPTX1 release into the synaptic cleft is confirmed (Farhy-Tselnicker et al. 2017), the perturbation of normal astrocytic functions—common feature of various neurodegenerative disorders (Phatnani and Maniatis 2015)—could harm the physiological function of NPTXs (Fig. 3b-1). The reduced BDNF levels described in several neurodegenerative disorders could lead to a diminished NPTX2 synthesis, as BNDF is one of the main regulators of NPTX2 (Lima Giacobbo et al. 2019; Mariga et al. 2015) (Fig. 3b-2). According to the level of neuronal activity, NPTXs modulate synaptic plasticity through structural changes in the post-synaptic membrane. Deregulation of neuronal activity could explain changes in NPTXs and drive neurogenerative processes (Xiao et al. 2017). Under low neuronal activity, NPTX1 contributes to neuronal death and neurodegeneration. Under those circumstances, NPTX1 is guided to the mitochondria, where it induces BAX-dependent apoptosis, allowing the release of pro-apoptotic factors (Clayton et al. 2012). Moreover, intracellular NPTX1 impairs mitochondrial translocation into the synapses—required due to the high energy demand—by reduction of anterograde trafficking (Clayton et al. 2012) (Fig. 3b-3). On the other hand, NPTX2 was originally described as an IEG induced by seizures and stroke in the rat hippocampus; mechanisms that involved an excess of glutamate in the synaptic cleft (Schwarz et al. 2002; Tsui et al. 1996). Excess of glutamate may lead to an activation of mGluR1/5 and the consequent TACE cleavage of NPTXR. The internalisation of AMPAR receptors upon TACE action (Cho et al. 2008) may serve as a protective mechanism against the excess of glutamate (Schwarz et al. 2002). Consequently, an impairment of this mechanism could contribute to the excitotoxicity observed in neurodegenerative disorders (Dong et al. 2009) (Fig. 3b-4). The recently described role of NPTX1 in synaptic pruning, by activation of the complement-microglia axis, could be intensified upon neurodegeneration (Kovács et al. 2020) (Fig. 3b-5). In summary, the deregulation of NPTXs expression levels, together with an imbalance in E/I and the alteration in their apoptotic roles, could explain the changes in NPTXs expression and their concentration in CSF and brain tissue in neurodegeneration. However, it raises the question of whether NPTXs actively contribute to neurodegeneration, or whether the deregulation of these proteins is a consequence of the synaptic damage.

### Neuronal pentraxins in neurodegenerative, psychiatric and other disorders of the CNS

#### Measurements of neuronal pentraxins in biological fluids

Measurements of NPTXs are mainly performed in CSF due to the insufficient assay sensitivity for a reliable quantification in blood samples. NPTXs levels can be quantified in CSF by means of mass spectrometry, enzyme-linked immunosorbent assay (ELISA) and Western Blot (WB) (Table 2). In comparison to those in other biological fluids, measurements of biomarkers in CSF offer several advantages. CSF is in direct contact with the brain, reflecting the changes occurring in this organ. Moreover, the dynamic range of CSF proteins is less variable, and CSF contains less high-abundant proteins that complicate the detection of low-expression proteins (Blennow et al. 2010). Additionally, the high expression of NPTXs in the brain compared with other body compartments reduces the risk of high peripheral NPTXs levels in the CSF. However, given that lumbar puncture is an invasive procedure, the need of CSF samples hinders the accessibility to longitudinal studies and the possibility to drive further conclusions regarding NPTXs potential as a disease progression marker. Accordingly, sensitive assays for reliable quantification in blood are needed. A study performed using WB was able to detect NPTXR in serum, and the values described the same trend as those obtained from CSF samples (Yin et al. 2009). This is reinforced by the correlation found between brain and plasma NPTX1 concentrations in transgenic AD mice (Ma et al. 2018). Hybrid immunoaffinity-mass spectrometry techniques have revealed the presence of synaptic proteins in blood (Kvartsberg et al. 2015; Oeckl et al. 2020), manifesting that NPTXs measurement in serum may be feasible in the future. However, the expression of some NPTX proteins in peripheral organs must be taken into consideration, as it could mask the pathological CSF findings in blood. The study of neuronal exosomes is a new promising research field to detect brain biomarkers in blood. Neuronal exosomes are released by neurons and carry cargo from their cells of origin. Due to their inherent capability to cross the blood–brain barrier, they are detectable in the blood (Song et al. 2020). A study on plasma neuronal exosomes of patients with AD dementia revealed declined levels of NPTX2 compared with matched controls. Similarly, its respective post-synaptic partner GluA4 already decreased in cognitively intact AD patients and further declined with disease progression, correlating with cognitive loss (Goetzl et al. 2018). However, the suitability of this method to specifically isolate plasma neuronal exosomes needs to be better clarified (Norman et al. 2021). In conclusion, novel, highly sensitive assays have to be established to further assess the potential of NPTXs as blood biomarkers in neurodegenerative disorders.

**Table 2:** Summary table of the studies assessing NPTXs CSF in Alzheimer’s disease (AD) and frontotemporal lobar degeneration (FTLD) included in the present review

	Study	Methodology	Study design	Cohort	Summary of findings		
					NPTX1	NPTX2	NPTXR
AD	Brinkmalm et al. (2018)	Mass spectrometry	Cross-sectional	15 CTL, 10 AD	↓ AD vs CTL		
	Xiao et al. (2017)	WB and ELISA	Cross-sectional	36 CTL, 30 AD	↓ AD vs CTL	↓ AD vs CTL	↓ AD vs CTL
	Duits et al. (2018)	Mass spectrometry	Longitudinal	40 CTL, 40 AD dementia, 40 MCI (13 sMCI, 14 MCI-AD)	↑ MCI vs CTL ↓ AD dementia vs MCI^a^		
	Galasko et al. (2019)	ELISA	Cross-sectional	90 CTL, 57 MCI, 46 AD		↓ MCI vs CTL ↓ AD vs CTL	
	Nilsson et al. (2021)	Mass spectrometry	Cross-sectional	Pilot study: 20 CTL, 20 AD Validation cohort: 20 CTL, 32 AD	≈ AD vs CTL	↓ AD vs CTL^b^	↓ AD vs CTL^b^
	Soldan et al. (2019)	ELISA	Cross-sectional	130 CTL, 19 MCI		↓ MCI vs CTL	
	Libiger et al. (2021)	Mass spectrometry	Longitudinal	76 CTL, 111 MCI		↓ 10% in 5 years in AD	
	Hendrickson et al. (2015)	Mass spectrometry	Longitudinal	30 CTL, 30 AD (severely impaired)			↓ AD vs CTL (↓ 6.9%/year)
	Lim et al. (2020)	ELISA	Longitudinal	46 CTL, 28 MCI, 27 MCI-AD, 28 AD			↓ AD vs CTL (↓ 6.7%/year)
	Llano et al. (2017)	Mass spectrometry	Cross-sectional	86 CTL, 135 MCI, 66 AD			↑ MCI vs CTL ↓ AD dementia vs MCI
	Wildsmith et al. (2014)	Mass spectrometry	Longitudinal	10 CTL, 5 MCI, 45 AD			↓ 10%/year in AD
	Begcevic et al. (2018)	Mass spectrometry and ELISA	Cross-sectional	Cohort MS: 8 MCI, 11 mild AD, 24 moderate AD, 15 severe AD Cohort ELISA: 6 MCI, 8 mild AD, 16 moderate AD, 13 severe AD			Progressive ↓ with disease severity
	Lim et al. (2019)	ELISA	Cross-sectional	14 MCI, 21 mild AD, 43 moderate AD, 30 severe AD			Progressive ↓ with disease severity
	Yin et al. (2009)	WB	Cross-sectional	4 CTL, 5 AD			↑AD vs CTL
FTLD	Van der Ende et al. 2019	Mass spectrometry	Cross-sectional	52 NC, 59 PMC, 99 SMC			↓ FTLD SMC vs NC
	Van der Ende et al. (2020)	ELISA and WB	Cross-sectional/ Longitudinal	70 NC, 106 PMC, 54 SMC	↓ FTLD SMC vs PMC and NC	↓ FTLD SMC vs PMC and NC. Progressive ↓ in SMC and in PMC over the age of 50 years	↓ FTLD SMC vs PMC and NC
	Barschke et al. (2020)	Mass spectrometry	Cross-sectional	28 asymptomatic C9orf72 MC, 18 c9FTD, 28 c9ALS			↓ c9FTD vs asymptomatic C9orf72 MC
	Remnestål et al. (2020)	Antibody suspension bead array	Cross-sectional	Pilot study: 8 NC, 16 PMC, 29 FTLD Validation cohort: 18 CTL, 79 AD, 13 FTLD			↓ FTLD vs CTL (NC, PMC)

^a^ NPTX1 was elevated in the patients with MCI-AD compared with sMCI. AD patients had lower, but not statistically significant, NPTX1 compared with CTLs. ^b^ No differences found in the pilot study (biochemically defined), only in the validation cohort (clinically diagnosed subjects). CTL, control; AD, Alzheimer's disease; MCI, mild cognitive impairment; sMCI, stable MCI; MCI-AD, MCI patients who progressed to AD; FTLD, frontotemporal lobar degeneration; NC, non-carriers; MC, mutation carriers; PMC, pre-symptomatic mutation carriers; SMC, symptomatic mutation carriers; c9FTD, FTLD cases with hexanucleotide repeat expansion in the C9orf72 gene; c9ALS, ALS cases with hexanucleotide repeat expansion in the C9orf72 gene

#### Alzheimer’s disease

AD is the most common type of dementia. It is pathologically characterised by the presence of extracellular plaques of amyloid β (Aβ), intracellular neurofibrillary tangles of tau protein and brain atrophy caused by neuronal loss, mainly in the entorhinal cortex, the hippocampus, the neocortex and the nucleus basalis (Henstridge et al. 2019). To date, CSF Aβ_42_, total tau and p-tau_181_ are the best characterised diagnostic fluid biomarkers in AD, reflecting the core pathologies described above (Blennow and Zetterberg 2018). However, there is a need for biomarkers that specifically address other pathological features of AD such as synaptic degeneration, which might have a better prognostic value and be better to evaluate the efficacy of new therapies. NPTXs may be potential candidate biomarkers in AD due to their expression in neocortex and hippocampus (Uhlén et al. 2015; Xu et al. 2003), areas that are largely affected by synaptic dysfunction and loss in AD (Davies et al. 1987; DeKosky and Scheff 1990; Reddy et al. 2005; Scheff et al. 2007). NPTXs might also underly some of the neurotoxic effects of Aβ aggregation (Abad et al. 2006), which is known to affect excitatory synaptic transmission and neuronal activity, mechanisms that precede neurite degeneration and neuronal death (Kamenetz et al. 2003; Walsh and Selkoe 2004) and subsequent cognitive failure (Driscoll and Troncoso 2011; Jack and Holtzman 2013). Accordingly, NPTXs were found to be the only markers that correlate with Aβ_42_ in AD (Nilsson et al. 2021). This could be due to a reduction in AMPAR (Rui et al. 2010; Xiao et al. 2017), glutamatergic receptors clustered and stabilised by NPTXs (O’Brien et al. 1999), thus resulting in a disruption of the pyramidal neuron-PV interneuron circuit (Xiao et al. 2017). PV interneurons, where NPTXs play a crucial role (Chang et al. 2010), are cells highly vulnerable in the AD brain (Brady and Mufson 1997; Takahashi et al. 2010). In a mouse model of Aβ amyloidosis, deficits in these interneurons contributed to abnormalities in oscillatory rhythms (especially in the gamma spectrum), network synchrony, and cognition (Verret et al. 2012). Optogenetically activated PV interneurons at gamma oscillations reduced Aβ_40_ and Aβ_42_ isoforms (Iaccarino et al. 2016). Last, the perineuronal net required for NPTX2 localization in the synaptic cleft (Chang et al. 2010) is affected in AD and might lead to the deregulation of this protein (Baig et al. 2005).

NPTX1 was upregulated in the brain of patients with sporadic late-onset AD, and located with Aβ and tau deposits in dystrophic neurites (Abad et al. 2006). By contrast, another study found no changes of NPTX1 in neither of the cortical regions analysed (hippocampus not included) (Xiao et al. 2017). NPTX1 may play a key role in synaptic loss, neurite damage and neuronal apoptosis, mechanisms evoked by Aβ (Abad et al. 2006). Several hypotheses could explain the role of NPTX1 in neuronal toxicity in AD. First, the translocation of NPTX1 from the synaptic cleft into the mitochondria could reduce the ability of the NPTX complex to cluster AMPARs and will activate BAX-dependent apoptosis (Clayton et al. 2012). Second, the increase in NPTX1 may promote the excessive synaptic loss in AD (Terry et al. 1991), due to an excess of the complement-mediated synaptic pruning (Kovács et al. 2020). Last, the interaction of NPTX1 with an intracellular form of NPTXR, capable of remodelling chromatin, may disrupt the association of NPTXR with the receptor protein tyrosine phosphatase O, required for neurite outgrowth (hypothesis under investigation) (Abad et al. 2006; Chen and Bixby 2005). Based on these data, reduction of NPTX1 expression could be a drug target to prevent synaptic dysfunction and loss in neurodegeneration (Figueiro-Silva et al. 2015). Several studies conducted in human CSF from AD patients reported a reduction of NPTX1 levels in AD patients compared with healthy controls (Brinkmalm et al. 2018; Xiao et al. 2017). On the other hand, NPTX1 levels were found to be elevated in patients with mild cognitive impairment (MCI) progressing to AD (Duits et al. 2018). The method of measurement (mass spectrometry vs WB) together with the small sample size could account for the discrepancy in the results. Furthermore, we could also speculate on an increase of CSF NPTX1 levels in the earliest stages of the disease, followed by a drop in the dementia phase (Table 2).

CSF NPTX2 levels assessed by WB, *in-house* ELISA and mass spectrometry were decreased in both sporadic and genetic forms of MCI and AD dementia in comparison with cognitive healthy controls (Belbin et al. 2020; Galasko et al. 2019; Nilsson et al. 2021; Soldan et al. 2019; Xiao et al. 2017). A mass spectrometry-based approach revealed NPTX2 as one of the best markers to differentiate between progressive and non-progressive MCI (Spellman et al. 2015). The receiver operating characteristic (ROC) analysis concluded a similar test performance to distinguish AD from controls than that of the classical AD biomarkers (Xiao et al. 2017). The ratio tau or p-tau with NPTX2 reported the best performance to discriminate AD vs controls (Galasko et al. 2019; Xiao et al. 2017). NPTX2 was also decreased in post-mortem cortex from AD patients (hippocampus not analysed) (Xiao et al. 2017). The subunit of AMPAR, GluA4, was also reduced in this study (both in the precuneus and medial frontal gyri) and correlated with NPTX2 in both control and AD group (Xiao et al. 2017). GluA4 is selectively enriched in PV interneurons in human cortex and is regulated by NPTXs (Sia et al. 2007; Xiao et al. 2017). Consequently, the reduction of NPTX2 could be linked to the disruption of pyramidal neuron-PV interneuron microcircuit, contributing to cognitive failure in AD (Xiao et al. 2017). This was further sustained by studies that found NPTX2 correlating with medial temporal atrophy, hippocampal volume and cognitive impairment (Nilsson et al. 2021; Swanson et al. 2016; Xiao et al. 2017). In addition, new evidence suggested NPTX2 as a strong prognostic biomarker candidate of cognitive decline (Libiger et al. 2021). Therefore, NPTX2 could be an accurate predictor of AD-outcome helping to track cognitive failure in AD (Galasko et al. 2019) (Table 2).

CSF NPTXR was identified by discovery proteomics to be associated with AD in several studies (Ringman et al. 2012; Wildsmith et al. 2014). CSF NPTXR levels, both the full length protein and the peptides resulting from TACE cleavage, were reduced in AD in comparison with controls (Xiao et al. 2017). Similar to NPTX1, NPTXR levels in CSF were slightly higher in MCI than in controls and decreased progressively with disease severity by an average of 7–10% per year (Hendrickson et al. 2015; Lim et al. 2020; Llano et al. 2017; Wildsmith et al. 2014). Accordingly, CSF NPTXR can discriminate between advanced AD stages and MCI (Area under the curve = 0.799) (Begcevic et al. 2018; Lim et al. 2019). These data were also consistent with a study in pre-symptomatic mutation carriers that reported a trend towards elevated NPTXR and NPTX2 CSF levels in comparison with non-carriers (Ringman et al. 2012). Another study documented higher levels of NPTXR in CSF of AD patients in comparison with controls. However, the severity of the cognitive impairment and the disease stage were not addressed (Yin et al. 2009). This, together with the method of measurement and the small sample size, could explain the differences observed. In summary, NPTXR could be a new biomarker of AD progression and help to assess therapy performance (Begcevic et al. 2018; Lim et al. 2020, 2019; Wildsmith et al. 2014) (Table 2).

Overall, these results reflect an increase of NPTX1 and NPTXR in pre-symptomatic stages of genetic AD, MCI and early stages of AD, whereas they decrease in later dementia stages (Duits et al. 2018; Lim et al. 2020; Llano et al. 2017; Ringman et al. 2012; Wildsmith et al. 2014). This is consistent with other studies that have suggested a transient peak in biomarker levels (neurosecretory protein VGF, neurexin, cystatin C, functional magnetic resonance imaging (MRI)) in the early stages of the disease (Dickerson et al. 2005; Duits et al. 2018; McDade et al. 2018). In addition, the behaviour of NPTX1 and NPTXR could be explained by the biphasic activity of the glutamatergic system in the hippocampus, with increased activity in MCI, followed by a decline during the dementia stage (Findley et al. 2019). By contrast, NPTX2 seems to be downregulated in all symptomatic stages of the disease, suggesting a different pathogenic behaviour of this protein in AD (Galasko et al. 2019; Nilsson et al. 2021; Soldan et al. 2019; Xiao et al. 2017). Taken together, NPTXs may be novel markers for AD, anticipating disease progression and predicting cognitive failure.

#### Frontotemporal lobar degeneration

FTLD is a group of progressive neurodegenerative disorders. It is characterised by a selective neuronal loss in the frontal and temporal lobes, leading to deficits in behaviour, executive function and/or language (Bright et al. 2019). The disease can be misdiagnosed as a psychiatric disorder due to the prominent behavioural symptoms (Bang et al. 2015; Rascovsky et al. 2011). To date, specific fluid diagnostic biomarkers are missing (Swift et al. 2021). Increasing evidence suggests synaptic degeneration to be one of the first pathological mechanisms in FTLD (Gong and Lippa 2010; Ling 2018; Lui et al. 2016; Marttinen et al. 2015). Therefore, synaptic biomarkers might contribute to early diagnosis and evaluation of disease progression. The impairment in hippocampal GABAergic function, the alteration in AMPAR and the reduction in the neurotransmitter glutamate in the frontal cortex (Gascon et al. 2014; Levenga et al. 2013; Murley et al. 2020; Procter et al. 1999) may lead to some of the neuropathophysiological characteristics of FTLD. Hence, NPTXs may be novel, possible candidate biomarkers given their role as regulators of AMPAR in PV GABAergic interneurons (Chang et al. 2010; O’Brien et al. 1999).

Recent studies have evaluated CSF NPTXs as novel biomarkers of genetic FTLD (Barschke et al. 2020; Remnestål et al. 2020; van der Ende et al. 2020, 2019). Data obtained with *in-house* ELISA exhibited lower CSF NPTX2 concentration in symptomatic mutations carriers than in pre-symptomatic and non-carriers. WB and mass spectrometry analysis of NPTX1 and NPTXR yielded similar results (van der Ende et al. 2020, 2019). NPTXs levels did not differ depending on genetic cause. The diagnostic accuracy of NPTX2 (Area under the curve: 0.71), to distinguish symptomatic mutation carriers from non-carriers, was comparable with that of neurogranin, one of the most accepted synaptic biomarkers (van der Ende et al. 2020). Among mutation carriers, NPTX2 correlated with cognitive impairment, grey matter volume of the whole brain as well as of frontal, parietal and temporal lobes and insula. A preliminary longitudinal analysis of 13 patients revealed a decline over time in NPTX2 in symptomatic mutation carriers and in pre-symptomatic mutations carriers over the age of 50 years (van der Ende et al. 2020). Furthermore, deep proteomic study by mass spectrometry revealed decreased NPTXR CSF levels in c9FTD compared with asymptomatic mutation carriers. c9FTD refers to the most common genetic form of FTLD that is caused by the hexanucleotide repeat expansion in the C9orf72 gene. Even though C9orf72 is also the cause of most of the genetic amyotrophic lateral sclerosis (c9ALS) cases, the decrease in NPTXR was specific to c9FTD (Barschke et al. 2020). To date, one study has reported data in sporadic FTLD by antibody suspension bead array. This study revealed decreased levels of NPTXR in CSF of FTLD patients (both sporadic and genetic forms) in comparison with non-carriers and pre-symptomatic mutation carriers. No differences in NPTXR CSF levels were detected between AD and FTLD (Remnestål et al. 2020) (Table 2).

In summary, these findings suggest an overall reduction of NPTXs in genetic and sporadic FTLD. In particular, NPTX2 decreases paralleling disease progression and may reflect an association between synaptic disruption and cognitive impairment. Moreover, NPTXR illustrates a different role of synaptic dysfunction in c9FTD and c9ALS, and their potential as differential diagnosis marker may be worth studying. Thus, the previous studies reinforce the potential application of NPTXs as an early disease and prognostic marker for FTLD, mainly in the genetic forms (van der Ende et al. 2020).

#### Synucleinopathies: Parkinson’s disease and dementia with Lewy bodies

PD and dementia with Lewy bodies (DLB) are proteinopathies characterised by aggregation of the pre-synaptic protein, α-synuclein (Camporesi et al. 2020; Outeiro et al. 2019; Wong and Krainc 2017). Synaptic dysfunction contributes to disease pathogenesis in PD (Nguyen et al. 2019) and may be linked to cognitive and motor dysfunction (Bereczki et al. 2017; Moran et al. 2008). NPTXs might be interesting biomarker candidates due to the high susceptibility of dopaminergic neurons in vitro to excitotoxicity and non-apoptotic cell death induced by the activation of AMPAR (regulated by NPTXs) (Dorsey et al. 2006). Moreover, NPTXs are expressed in hypothalamic nerve cells, which may be implicated in the sleep disturbances found in PD patients (Crocker et al. 2005; Thannickal et al. 2007). A whole genome expression profiling revealed NPTX2 to be the most highly upregulated gene in the substantia nigra of sporadic PD patients (Moran et al. 2008). Additionally, it accumulated in Lewy bodies and neurites in substantia nigra and cerebral cortex (Moran et al. 2008). NPTX2 may be implicated in the motor and cognitive impairment caused by dopaminergic nerve cell degeneration in the midbrain and synaptic alteration in the cerebral cortex, respectively (Lang et al. 2020; Moran et al. 2008). In a mouse model of PD, NPTX2 was overexpressed in the striatum after L-DOPA treatment and contributed to the development of L-DOPA-induced dyskinesia (Charbonnier-Beaupel et al. 2015). Synaptic dysfunction and loss, mainly localised in the primary visual cortex, may also contribute to DLB (Khundakar et al. 2016; Mukaetova-Ladinska et al. 2013). This could be due to an impairment of the GABAergic transmission and a possible interneuron dysfunction in this region (Khundakar et al. 2016), where NPTXs participate in synaptic refinement (Bjartmar et al. 2006). NPTX2 CSF levels were lower in patients with DLB than in cognitive healthy controls (Boiten et al. 2020). Additionally, these values correlated with cognitive function in the visual spatial domain (Boiten et al. 2020). These results suggested an impairment of the principal neuron-interneuron microcircuit in the primary visual cortex in DLB patients, reflected by a decline in CSF NPTX2 levels (Boiten et al. 2020). Further to this, NPTXR and NPTX1 were decreased in atypical parkinsonian disorders (progressive supranuclear palsy, multiple system atrophy, corticobasal degeneration) in comparison with controls (Magdalinou et al. 2017). To summarise, the results stated above support NPTX2 as a novel candidate protein in α-synuclein pathology.

#### Psychiatric disorders

Dysfunction and/or deficient inhibitory output from PV interneurons may be a common mechanism of multiple psychiatric disorders, such as schizophrenia (SCZ) (Lewis et al. 2005), autism spectrum disorders (ASD) (Gao and Penzes 2015) and bipolar disorder (BD) (Ruden et al. 2021). The disruption of the perineuronal net, required for NPTX2 localization in the synaptic cleft (Chang et al. 2010) and disrupted in SCZ, might be contributing to the previously described GABAergic dysfunction (Berretta et al. 2015). In vivo studies suggested improvements in cognition and memory by activation of PV interneurons in the medial prefrontal cortex, restoring GABAergic signalling and E/I balance (Ferguson and Gao 2018). In addition, an imbalance in synaptic connectivity during perinatal and adolescence periods is being hypothesized as a possible mechanism of SCZ and ASD (Gilbert and Man 2017; McGlashan and Hoffman 2000). Hence, we speculate an involvement of NPTXs in the pathogenic mechanism of psychiatric disorders given their role in synaptogenesis, synaptic pruning and in the regulation of the excitatory drive onto PV interneurons. Chromosomal breakpoint analysis in patients with BD suggested NPTX1 as a novel candidate gene for BD (Rajkumar et al. 2015). NPTX1 could contribute to BD pathology by deregulation of glutamatergic synaptic transmission (Eastwood and Harrison 2010).

#### Other disorders of the CNS: epilepsy, stroke, neuroinflammatory diseases and brain tumours

NPTXs have also been studied in other disorders affecting the CNS. NPTX2 was initially discovered to be released from the pre-synaptic terminal in response to seizures (O’Brien et al. 1999). In accordance with these results, *Nptx2* mRNA was enriched in the rat hippocampus after maximal electroconvulsive seizure (MECs) (Tsui et al. 1996). However, NPTX1 was constitutively expressed in the adult brain with levels unchanged after MECs (Xu et al. 2003). Aside from these initial discoveries, little research has focussed on the role of NPTXs in epileptic seizures. NPTXs could contribute to the long-term E/I imbalance characteristic of epilepsy by altering AMPAR neurotransmission (Bonansco and Fuenzalida 2016; Lopes et al. 2013). The role of NPTXs was also explored in ischaemic stroke. NPTX1 was induced after hypoxic-ischaemic neuronal injury and led to the release of cytochrome C and the activation of caspase-3 (Rahim et al. 2013). In in vitro conditions that mimic human strokes, the deletion of NPTX1 resulted in decreased neuronal death in comparison with wildtype neurons, potentially due to the reduced surface GluA1. This indicates a crucial role of NPTX1 in AMPAR clustering and suggests NPTX1 as a potential target to prevent the consequences of strokes (Al Rahim and Hossain 2013). Similarly, NPTX2 was upregulated in a rat model of cerebral ischaemia. This may promote AMPAR internalization, protecting against excitotoxicity (Schwarz et al. 2002). There is limited evidence to sustain the role of NPTXs in neuroinflammatory disorders. One study reported decreased CSF levels of NPTXR in multiple sclerosis compared with other neurological disorders (Kroksveen et al. 2012). Hence, future studies need to address the potential of these proteins as biomarkers of neuroinflammation. The involvement of NPTXs was broadly studied in brain cancer research (Bartolini et al. 2015; Huo et al. 2019; Wang et al. 2020). Notably, the involvement of these proteins in tumour development might be related to the role of AMPAR in tumours (Radin and Patel 2017; Wang et al. 2020), together with the inflammatory function of NPTXs. However, in-depth research is needed to determine their involvement in tumour progression.

#### Correlation between NPTXs and other recognised markers of neurodegeneration

Several studies addressed the correlation between NPTXs and already established biomarkers. NPTX2 exhibited a weak correlation with p-tau_181_ and total tau (Xiao et al. 2017). With total tau, the correlation was more robust in the late stages of AD (Galasko et al. 2019). On the other hand, the Aβ_42_ data is less consistent. Some authors described a weak correlation with Aβ_42_, mainly in MCI (Galasko et al. 2019; Nilsson et al. 2021), whereas others found no correlation (Xiao et al. 2017)*.* NPTX1 and NPTXR did not correlate with Aβ_42,_ but did weakly correlate with total tau and p-tau_181_ (Xiao et al. 2017)_._ Another study reported no correlation between NPTXR and core AD biomarkers, except in the control group (Lim et al. 2020). The authors suggested the biphasic behaviour of NPTXR in CSF could explain these results across the different stages of AD (Lim et al. 2020)*.* NPTXs seemed to moderately correlate with many synaptic markers (e.g., β-synuclein, γ-synuclein, neurogranin, syntaxin, SNAP25) in the healthy control and the AD group. Intriguingly, they declined in AD patients, opposite to the other synaptic markers that primarily increased in CSF (Galasko et al. 2019; Nilsson et al. 2021). As for non-AD conditions, NPTX2 correlated with CSF levels of other synaptic proteins such as neurosecretory protein VGF and α-synuclein in DLB (Boiten et al. 2020). Moreover, NPTX2 was inversely correlated with CSF neurofilament light concentrations—a marker of neuroaxonal damage and disease severity—in genetic forms of FTLD (van der Ende et al. 2020). Finally, CSF levels of the different NPTXs strongly correlated with each other in both AD and genetic FTLD, indicating an overall reduction of these proteins in CSF (Nilsson et al. 2021; van der Ende et al. 2020; Xiao et al. 2017).

## Discussion and conclusion

The progressive synaptic impairment in neurodegenerative and other diseases of the CNS remains to be fully elucidated. However, emerging scientific literature supports its role during the first stages of neurodegeneration. More research needs to be conducted to reveal the pathological mechanisms that lead to synaptic degeneration and loss. Untangling the pathological changes occurring in the first steps of these diseases could provide critical information for early diagnosis and even help to identify novel therapeutic targets.

As synaptic proteins, NPTXs represent promising early disease and prognostic biomarker candidates for structural and functional synaptic impairment in neurodegenerative conditions. Unlike core biomarkers such as Aβ (Williams et al. 2011), CSF NPTXs levels correlated with cognitive scores in AD patients, even in the absence of brain pathology. Thus, they might be a good candidate biomarker for disease staging and cognitive prognosis (Galasko et al. 2019; Libiger et al. 2021; Nilsson et al. 2021; Swanson et al. 2016; Xiao et al. 2017). NPTXs also showed an added role when combined with core AD biomarkers in predicting the progression from MCI to AD dementia and in discriminating AD and healthy controls, e.g., NPTX2 in ratio with either total tau or p-tau (Galasko et al. 2019; Xiao et al. 2017). However, new studies addressing the value of CSF NPTXs in the differential diagnosis between AD and other neurodegenerative disorders are needed.

NPTXs may contribute to the cognitive decline in neurodegeneration and other diseases of the CNS by modifying the excitatory synaptic drive onto fast-spiking PV interneurons which leads to E/I imbalance. This might promote or accelerate disease progression by increasing the demand in other compensatory mechanisms (Hu et al. 2010; Shepherd et al. 2006; Xiao et al. 2017). In addition, NPTXs deregulation could lead to an imbalance in synaptogenesis/synaptic pruning (Kovács et al. 2020). The AD studies displayed in this review describe a tendency towards a transient increase in NPTXs during early stages of the disease, when the cognitive impairment is mild, followed by a progressive decrease paralleling disease severity. One potential explanation is the existence of a compensatory mechanism in the early stages of the synaptic damage, followed by a decrease in synaptic markers due to neuronal loss. In the pre-symptomatic stage, NPTXs may act as a resilience factor keeping the integrity of the synapses, as previously hypothesised (Xiao et al. 2017). When the disease progresses, the loss of NPTXs may contribute to the cognitive impairment. Another hypothesis is that the increased CSF synaptic protein concentration may be a consequence of the protein leakage into the interstitial fluid upon synaptic damage and early neuronal loss (Wellington et al. 2016); whereas in later stages, the aggregation with misfolded proteins in the tissue (Bereczki et al. 2017) and the confounded widespread neurodegeneration would explain the reduced CSF values. Moreover, the role of NPTX1 in neuronal cell death could also explain the increased levels of this protein in early stages of the disease. A validation of the results obtained in the FTLD cohort is required to confirm a similar behaviour of NPTXs in this pathology. The increased NPTX1 observed in brain tissue from AD patients may be due to the aggregation into neurite plaques. Taken together, the disease stage might affect the synaptic protein levels both in the brain and in CSF.

The application of NPTXs as fluid biomarkers in neurodegeneration requires to overcome some outstanding challenges. First, to unravel their signalling pathways, role in the disease and behaviour in the biofluids. This would help to clarify the sometimes-controversial levels of the different NPTXs in CSF and brain tissue. Second, to untangle the detailed expression pattern of these proteins in the different regions of the brain. Third, to standardise protocols regarding sample handling, methodology and analytical parameters to get reproducible data. Fourth, to get access to big cohorts with patients that have been well characterised clinically and to define a clear inclusion and exclusion criteria. This would allow further conclusions regarding the behaviour of these proteins during the course of the diseases. Fifth, to analyse the levels of CSF NPTXs across a broader range of diseases of the CNS, including both genetic and sporadic subtypes. Last, to address the potential of these proteins as markers of disease progression, moving from cross-sectional to longitudinal study designs. The latest improvements in high-resolution mass spectrometry, together with automated immunoassays and single molecule array (Simoa), may allow the development of more sensitive assays capable of measuring NPTXs and other synaptic markers both in CSF and blood. In addition, new studies are needed to determine the potential of plasma neuron-derived exosomes as a new approach to detect early biomarkers of neurodegeneration in blood samples (Song et al. 2020).

Additional questions remain to be addressed to understand the role of NPTXs in synaptic function and their potential as fluid biomarkers and drug targets. Is the deregulation of NPTXs one of the pathological mechanisms behind cognitive impairment in neurodegenerative and psychiatric disorders, or is it merely a consequence of the synaptic damage? Do NPTXs participate in the PV interneurons impairment observed in various diseases of the CNS? Considering the structural similarity between NPTXs and the classical pentraxins CRP and SAP, could these proteins play a complementary role in synaptic activity and neuroinflammation during blood–brain barrier breakdown (Cummings et al. 2017)? Could NPTXs participate in pathological neuroinflammation? Is their role as mediators of the complement-dependent synaptic pruning, altered upon pathological conditions, explaining the excess of neuronal loss in neurodegenerative diseases? While NPTX2 promotes synaptic plasticity at excitatory synapses, NPTX1 is part of the mechanism of neuronal apoptosis and negatively regulates synaptic plasticity at excitatory synapses (DeGregorio-Rocasolano et al. 2001; Figueiro-Silva et al. 2015; Tsui et al. 1996). This “opposite” molecular mechanism could act as a homeostatic feedback and may have important implications in the role of NPTXs in neurodegenerative disorders. The synaptic changes occurring even a decade before clinical onset reflect the potential applicability of synaptic proteins as therapeutic targets (Ringman et al. 2012). Innovative molecular tools intended to restore the function of synaptic organizers, as NPTXs, could provide potential treatments to remodel impaired neuronal circuits in neurological disorders (Suzuki et al. 2020). Further studies are required to determine NPTXs potential as in vivo monitoring of synaptic dysfunction and drug targets.

In summary, excess synaptic loss and alteration of the post-synaptic terminal are common mechanisms of neurodegeneration. NPTXs may contribute or reflect the impairment in synaptic plasticity and/or loss by altering the normal integration of AMPAR in the post-synaptic terminal and disrupting the synaptogenesis/synaptic pruning balance. They are a promising candidate for early diagnosis and prognosis of cognitive decline and may provide crucial information to understand some of the pathological mechanisms behind many diseases of the CNS. However, the observed alteration in different disorders reflect a general pathological pattern and not a disease-specific mechanism. Thus, their potential suitability for clinical diagnostic purposes requires further investigation. The development of new assays for the detection of neurodegenerative processes is crucial to prevent misdiagnosis and to accelerate diagnosis upon onset of symptoms.
